# Evaluation of COVID-19 Diagnosis Codes for Identification of SARS-CoV-2 Infections in a Nursing Home Cohort, 2022–2023

**DOI:** 10.1016/j.jamda.2024.105440

**Published:** 2025-01-18

**Authors:** Arshiya Patel, Amanda B. Payne, Dustin W. Currie, Thomas Franceschini, Amber Gensheimer, Joseph D. Lutgring, Sujan C. Reddy, Kelly M. Hatfield

**Affiliations:** a Division of Healthcare Quality Promotion, National Center for Emerging and Zoonotic Infectious Diseases, Centers for Disease Control and Prevention, Atlanta, GA, USA; b Professional Services Business Unit, Chenega Enterprise, Systems, and Solutions, Anchorage, AK, USA; c Coronavirus and Other Respiratory Viruses Division, National Center for Immunizations and Respiratory Diseases, Centers for Disease Control and Prevention, Atlanta, GA, USA; d Signature Healthcare, Louisville, KY, USA

**Keywords:** COVID-19, SARS-CoV-2, ICD-10, nursing home, electronic health record, respiratory infections

## Abstract

**Objectives:**

This study aimed to evaluate the utility of electronic health record (EHR) diagnosis codes for monitoring SARS-CoV-2 infections among nursing home residents.

**Design:**

A retrospective cohort study design was used to analyze data collected from nursing homes operating under the tradename Signature Healthcare between January 2022 and June 2023.

**Setting and Participants:**

Data from 31,136 nursing home residents across 76 facilities in Kentucky, Tennessee, Indiana, Ohio, North Carolina, Georgia, Alabama, and Virginia were included.

**Methods:**

Resident demographics, diagnosis codes associated with clinical diagnoses (including COVID-19), and SARS-CoV-2 testing information were collected from the EHR and supplemental testing data sources. We described the rates of infection and the clinical characteristics of residents with incident-positive SARS-CoV-2 tests and new-onset COVID-19 diagnoses. Positive predictive values (PPVs) of COVID-19 diagnosis codes were calculated for residents stratified by whether a resident was continuously present in a facility for ±3 days from the diagnosis onset date listed in EHRs, using positive SARS-CoV-2 tests to confirm infection.

**Results:**

A total of 4876 incident-positive SARS-CoV-2 tests and 6346 new-onset COVID-19 diagnoses were recorded during the study period. Weekly rates of new-onset diagnoses were significantly higher than positive test rates, although trends followed similar trajectories. Among residents continuously present in the nursing home ±3 days from the diagnosis onset date, the PPV of COVID-19 diagnosis codes was high (3395 of 3685 = 92%; 95% CI, 91%–93%). The PPV among this group significantly varied by study quarter (*P* <.001). The PPV was substantially lower for 2661 diagnoses among residents not continuously present in the nursing home (24%; 95% CI, 22%–26%).

**Conclusions and Implications:**

This study demonstrates the utility of diagnosis codes for assessment of COVID-19 epidemiology and trends when testing data are unavailable for residents during their stay in a nursing home. Future research should explore strategies to evaluate the utility of diagnosis codes at admission and discharge to nursing homes to enhance surveillance efforts.

Coronavirus disease 2019 (COVID-19) has disproportionately affected nursing home residents.^1-3^ Given their immunosenescence, congregate living arrangements, presence of comorbidities, and other medical complexities, older adults residing in nursing homes are substantially more vulnerable to COVID-19–related morbidity and mortality compared with their counterparts living in a community setting.^4^ Thus, monitoring trends of COVID-19 in the nursing home setting is important.

SARS-CoV-2 infections in nursing home settings have been required by the Centers for Medicare and Medicaid Services (CMS) to be monitored using the National Healthcare Safety Network (NHSN) COVID-19 Long-term Care Facility Module since May 2020.^5,6^ NHSN does not require reporting of detailed resident characteristics, such as comorbidities, that allow for robust studies to assess outcomes of infections and vaccine effectiveness.^7^ Electronic health record (EHR) data include clinical diagnoses recorded on a nursing home resident’s problem list using International Classification of Diseases, 10th Revision, Clinical Modification (ICD-10-CM) codes with a corresponding diagnosis onset date. Codes from EHR data may provide detailed information on resident COVID-19 diagnoses and comorbidities. However, EHR data often do not include standardized or complete laboratory test information, particularly from point-of-care testing which makes assessment of laboratory-confirmed diagnoses of SARS-CoV-2 infections in EHR data difficult.^8^

Analyses of data systems that include COVID-19 diagnosis codes from EHR problem lists and testing information can assess the effectiveness of using these codes as markers of COVID-19 infections in nursing home residents when SARS-CoV-2 testing data are unavailable. In the first year of the pandemic, 93% of individuals with a positive SARS-CoV-2 test were shown to have at least 1 COVID-19 diagnosis code in their nursing home EHR problem list, with high correlation (r = 0.99) between their first positive SARS-CoV-2 test date and the first diagnosis date.^8^ However, concordance decreased over time, with only 61% of positive cases having the COVID-19 code from March to May 2021 compared with 97.5% in the same months in 2020.^8^ Our study aimed to evaluate the utility of using COVID-19 diagnosis codes in a nursing home resident’s EHR problem list for identifying SARS-CoV-2 infections in the nursing home.

## Methods

### Data Sources

In this retrospective cohort study, data from nursing homes operating under the tradename Signature Healthcare were collected to ascertain resident census, clinical diagnoses, and specimen collection dates and results from SARS-CoV-2 tests administered within the nursing home for any resident present in a participating nursing home from January 1, 2022, through June 30, 2023. Data were accessed on August 11, 2023.

Daily census records were used to identify which residents were present in each nursing home each day throughout the study period. EHR data were used to describe resident characteristics including sex, age, history of COVID-19, number of comorbidities, and length of stay. All ICD-10-CM diagnoses and the corresponding diagnosis onset date were captured from the EHR problem list, recorded using standard of care procedures (ie, at admission and throughout the nursing home stay), and categorized by quarter in our study period. The number of comorbid conditions (including cancer, chronic kidney disease, chronic obstructive pulmonary disease, heart conditions, immunocompromised state, chemotherapy, radiation, obesity, sickle cell disease, smoking, and diabetes) were identified by the presence of diagnosis codes in the EHR (Supplementary Table 1). SARS-CoV-2 supplemental testing data included all nursing home–administered SARS-CoV-2 tests since January 1, 2021 (which includes historical testing information) from comprehensive nursing home records. Tests were categorized using specimen collection date by quarter. Resident length of stay was categorized as short-stay for residents who were present for 90 days or less in the nursing home in our study period and long-stay for residents who were present for more than 90 days in our study period. For each incident-positive test or new-onset COVID-19 diagnosis, we identified previous COVID-19 infections by denoting the most recent COVID-19 diagnosis onset date or a positive SARS-CoV-2 test with a specimen collection date that was recorded more than 15 days prior.

### Definitions and Infection Rates

We assessed the overall and weekly COVID-19 rates per 1000 resident-days using 2 definitions for the entire study period. The first definition was test-based and required an incident-positive SARS-CoV-2 viral test in a resident without a positive test in the previous 14 days. The second definition was code based and required a new-onset COVID-19 diagnosis in the EHR problem list. We included diagnoses for U07.1 (COVID-19 infection) or J12.82 (pneumonia due to COVID-19) without any other COVID-19 diagnosis onset dates in the previous 14 days to determine the code-based rate. We compared the weekly test-based and code-based rates using a paired-wise t-test.

### Positive Tests for SARS-CoV-2 Infection

We described incident SARS-CoV-2–positive tests by resident characteristics overall and stratified by the presence of a corresponding COVID-19 diagnosis code. Significant associations between resident characteristics and the proportion of tests with corresponding diagnosis codes were assessed using χ^2^ statistics.

### COVID-19 Diagnosis Codes

We described characteristics of new-onset diagnoses. To account for potential misclassification due to positive tests being collected outside the nursing home (and therefore not available in the nursing home documentation), we stratified analyses of new-onset diagnoses into 2 groups: residents who were continuously present for ±3 days from the diagnosis onset date and those who were not continuously present during that time period, according to the daily resident census records. We expected residents who were present for the 3 days before and after their diagnosis onset date to have been diagnosed in the nursing home for that infection, as opposed to an outside setting. For continuously present residents, significant associations between resident characteristics and the proportion of diagnoses with a corresponding positive test were assessed using χ^2^ statistics.

We used a positive SARS-CoV-2 viral [ie, antigen and polymerase chain reaction (PCR)] test documented in the nursing home and provided supplemental testing data as confirmation of infection to calculate the positive predictive value (PPV) of a COVID-19 diagnosis code (ie, the proportion of all new-onset COVID-19 diagnosis codes that have a corresponding positive test with a specimen collection date ±7 days from the diagnosis onset date). We described PPV among continuously present residents by resident characteristics. We calculated the proportion of incident-positive SARS-CoV-2 tests (as the denominator) with a concurrent new-onset diagnosis code in the EHR problem list (as the numerator). For all PPV and concordance estimates, 95% CIs were calculated using a normal approximation for a binomial proportion. For significance testing, we used alpha = 0.05. All analyses were done using SAS v9.4.

This activity was reviewed by the Centers for Disease Control and Prevention (CDC), deemed not research, and was conducted consistent with applicable federal law and CDC policy (see eg, 45 CFR. part 46.102(l) (2)).

## Results

Our cohort included 31,136 distinct nursing home residents present for a total of 3,278,584 resident-days from 76 nursing homes in Kentucky (40 homes), Tennessee (21), Indiana (4), Ohio (4), North Carolina (3), Georgia (2), Alabama (1), and Virginia (1). Nursing homes had a median average daily census of 78 residents [Quartile 1 to Quartile 3 (Q1–Q3): 66–90] and a median of 108 licensed beds (Q1–Q3: 95–138).

### Positive Tests for SARS-CoV-2 Infection

During our study period, 4143 of 4876 (85%; 95% CI, 84%–86%) incident-positive SARS-CoV-2 viral tests had a corresponding COVID-19 diagnosis code within ±7 days of the positive test (Table 1, Supplementary Figure 1). Positive tests were most common from January to March 2022 (n = 1870, 38% of all positive tests, Table 1) and least common from April to June 2023 (n = 172, 4%). Two-thirds of positive tests were among long-stay residents (n = 3306, 68%, Table 1). Overall, three-fourths of tests were the first nursing homeedocumented occurrence of COVID-19 for the resident (n = 3718, 76%, Table 1). Among incident-positive SARS-CoV-2 tests, the proportion having a corresponding diagnosis code within ±7 days was significantly associated with quarter of specimen collection and previous diagnosis (*P* < .001 for both, Table 1).

### COVID-19 Diagnosis Codes

Throughout our study period, 6346 new-onset COVID-19 diagnoses were recorded in the EHR problem list (Table 2, Supplementary Figure 1). New-onset COVID-19 diagnoses occurred most frequently from January to March 2022 (n = 2,408, 38%) and least frequently from April to June 2023 (n = 237, 4%), consistent with positive tests. Almost half of new diagnoses (n = 3246, 51%) occurred among long-stay residents, and 84% were the first listed occurrence of COVID-19 for residents (n = 5355). Of all new-onset COVID-19 diagnoses, 2661 (42%) occurred among residents who were not present for at least ±3 days from their diagnosis onset date. Among these, only 628 had a corresponding positive SARS-CoV-2 test result reported in the nursing home for a PPV of a COVID-19 diagnosis being confirmed with a positive SARS-CoV-2 test of 24% (95% CI, 22%–26%). Most new-onset diagnoses for non–continuously present residents occurred among residents categorized as short-stay (n = 2149 of 2661, 81%, Table 2), whereas most new-onset diagnoses for continuously present residents were among residents categorized as long-stay (n = 2734 of 3685, 74%, Table 2). Further, 96% of new-onset diagnoses were the first documented COVID-19 infection among non–continuously present residents (n = 2549), compared with only 76% (n = 2806) among continuously present residents (Table 2).

### Infection Rates

**Fig. 1.**
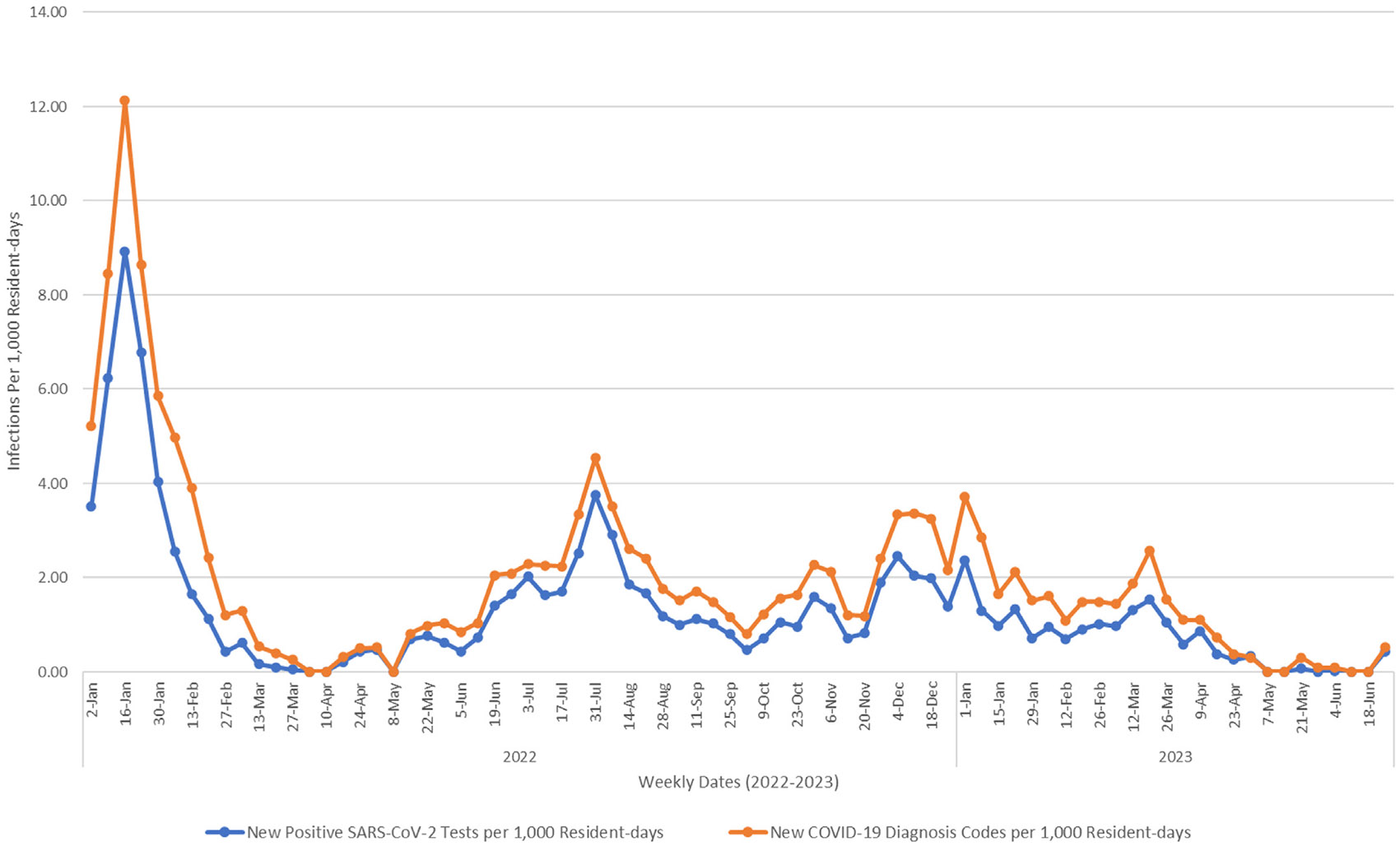
Weekly rates of new-onset COVID-19 diagnoses and SARS-CoV-2–positive tests per 1000 resident-days among 76 nursing homes, January 2022–June 2023.

The overall rates of new-onset COVID-19 diagnosis codes and incident-positive SARS-CoV-2 tests were 1.94 and 1.49 per 1000 resident-days, respectively. As shown in Figure 1, weekly incident SARS-CoV-2 infection rates per 1000 resident-days (defined using incident-positive SARS-CoV-2 tests) were lower than new-onset COVID-19 diagnosis rates (defined using the EHR problem list); the code-based rate was significantly higher than the test-based rate (*P* < .001). Both rates showed similar trends throughout the study period, with the highest rates occurring during the week ending January 23, 2022 (week 3, Figure 1).

### PPV of COVID-19 Diagnosis Codes Among Continuously Present Residents

Of 3685 new-onset COVID-19 diagnoses among continuously present residents, 3395 had a corresponding positive test resulting in a PPV of 92% (95% CI, 91%–93%); characteristics and test PPV are shown in Table 3. Of the 290 (8%) continuously present residents with a new-onset COVID-19 diagnosis code but without a corresponding positive test, 150 (52%) were administered a SARS-CoV-2 test within ±7 days of the diagnosis onset date, whereas 140 (48%) had no test recorded. The PPV of diagnosis codes being confirmed with a positive SARS-CoV-2 test varied significantly by quarter of the study period; the PPV was higher in April to June 2022 (96%; 95% CI, 94%–99%) and July to September 2022 (98%; 95% CI, 97%–99%), and lower in January to March 2023 (88%; 95% CI, 85%–90%). The PPVs of a COVID-19 diagnosis being confirmed with a positive SARS-CoV-2 test were similar for short-stay (93%; 95% CI, 91%–94%) and long-stay residents (92%; 95% CI, 91%–93%) and by number of assessed comorbid conditions a resident had documented in the EHR. Diagnoses from residents with a history of COVID-19 more than 180 days before or between 90 and 180 days before the diagnosis onset date had a similar PPV of a COVID-19 diagnosis being confirmed with a positive SARS-CoV-2 test to diagnoses from residents with no history of COVID-19. In contrast, the 1% of diagnoses from residents with a history of COVID-19 in the 15 to 90 days before the diagnosis date had a distinctively lower PPV (15%; 95% CI, 0%–32%).

**Fig. 2.**
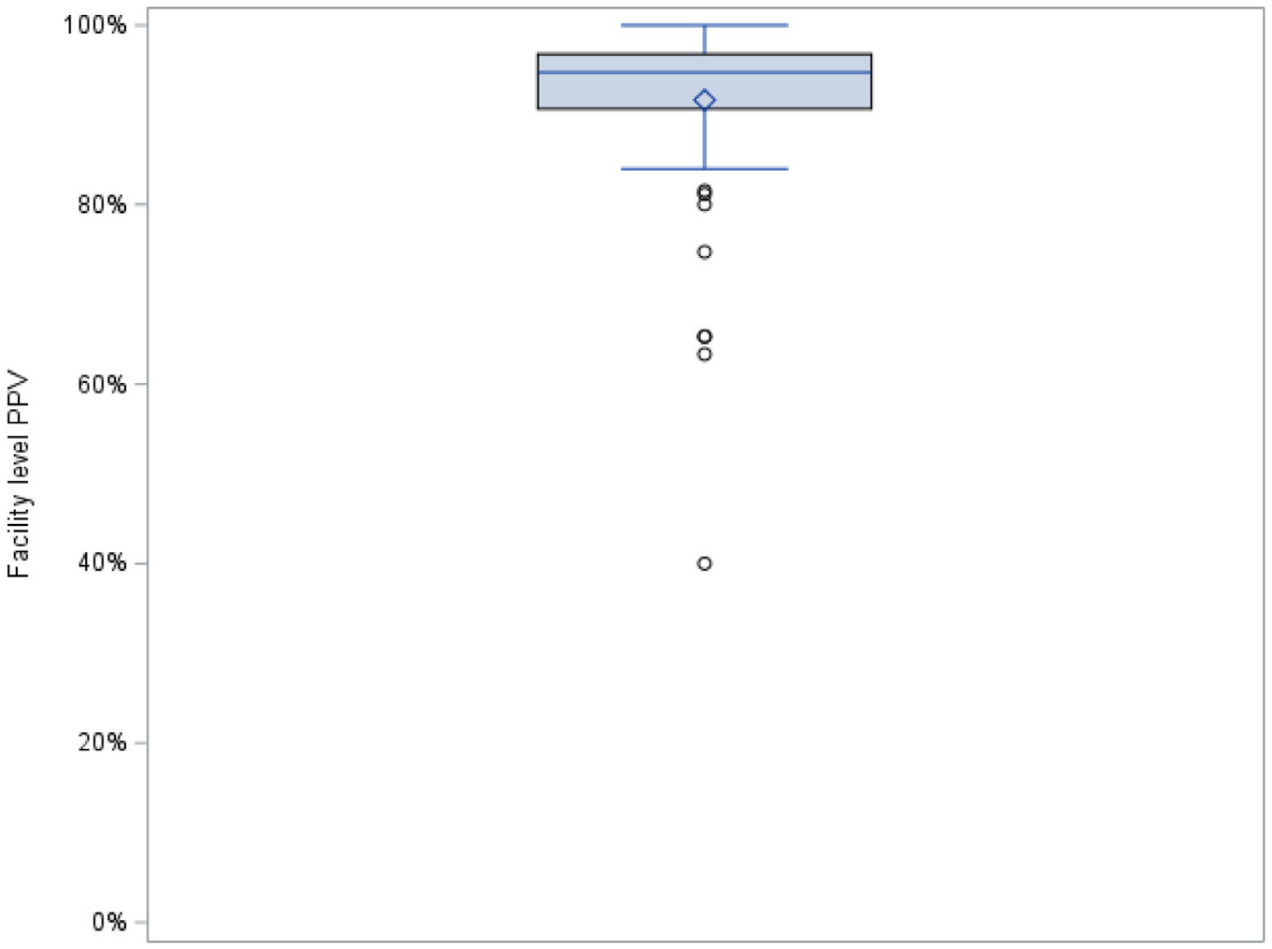
Variability of the PPV of diagnosis codes being confirmed with a positive SARS-CoV-2 test for continuously present residents in 76 nursing homes. PPV was defined as the proportion of all new-onset COVID-19 diagnosis codes with a corresponding positive test within ±7 days from the diagnosis onset date and is shown only for residents who were continuously present for ±3 days from the diagnosis onset date. In the box plot, the diamond-shaped marker inside the box indicates the mean value; the horizontal line inside the box indicates the median value; the bottom and top edges of the box indicate the intraquartile range (IQR), that is, the range of values between the first and third quartiles (the 25th and 75th percentiles); the top and bottom of the vertical line show the maximum (75th percentile value + 1.5 × IQR) and minimum (25th percentile value − 1.5 × IQR); and the open circles represent outliers for each distribution.

By nursing home, the PPV for diagnosis codes among continuously present residents ranged from 40% to 100%, with a median of 95%; PPV was greater than 90% in 58 of 76 nursing homes (76%, Figure 2).

## Discussion

In this study of a large cohort of more than 30,000 nursing home residents from 76 facilities spanning multiple states and 1.5 years, we demonstrated that the rates of COVID-19 using new-onset diagnosis codes from the EHR problem list and positive SARS-CoV-2 testing data showed similar changes over time. Infection rates using EHR diagnosis codes were consistently higher than rates using testing data. The PPV of diagnosis codes compared with a gold standard of a positive test for residents who were continuously present was high, but use of diagnosis codes for COVID-19 in the EHR to monitor SARS-CoV-2 laboratory-confirmed infections did not perform well among residents who were not continuously present in the nursing home for ±3 days from their diagnosis onset date. We showed 85% of incident-positive SARS-CoV-2 tests had a concurrent new-onset diagnosis code in the EHR problem list.

The number of new-onset COVID-19 diagnoses and incident-positive tests for SARS-CoV-2 varied significantly throughout the study period, with more than one-third of all cases being identified from January to March 2022 and less than 5% being identified in April to June 2023. These rates are likely influenced by differing levels of SARS-CoV-2 virus circulating in the nursing home and various intensities of testing practice during different time periods throughout our study. The PPV was highest from July to September 2022 (98%) and lowest (86%) in the final quarter of our study period, indicating potentially lower concordance in more recent months. The PPV of diagnosis codes was lower in the 1% of all new diagnoses with a prior COVID-19 diagnosis in the past 15 to 90 days, potentially because residents with recent repeat positive tests were not considered by the clinical team to have new-onset infections. Importantly, there were no discernable differences between the PPV of diagnosis codes for residents without a history of COVID-19 compared with residents with a history of COVID-19 more than 90 days ago, suggesting diagnosis codes may be used to identify repeat infections 90 or more days apart in nursing home residents.

Previous studies have demonstrated high concordance between COVID-19 diagnosis codes in administrative claims data and positive SARS-CoV-2 tests. A large retrospective cohort study found that the U07.1 code exhibited a sensitivity of 81% in outpatient claims and 94% in inpatient claims, compared with a gold standard of laboratory-confirmed positive tests.^9^ In addition, diagnosis codes demonstrated a PPV of 93% in outpatient and 95% in inpatient claims for identifying laboratory-confirmed COVID-19 cases.^9^ Similarly, studies of inpatient claims and electronic health records found that COVID-19 diagnosis codes had a PPV of more than 90% when compared with the reference standard of a SARS-CoV-2 PCR positive test.^10-14^ Although most nursing home residents in the United States are covered by Medicare insurance, only a subset of short-stay nursing home residents receiving post-acute care can submit Medicare claims to pay for their stay. Thus, unlike the inpatient or outpatient setting, Medicare claims-based analyses for nursing home infection surveillance are likely incomplete due to complicated billing structures in nursing homes.

Surveillance systems, such as NHSN, provide real-time monitoring of trends of COVID-19 in nursing homes.^15^ However, NHSN does not collect detailed information on uninfected residents, resident comorbidities, or prior infections. These resident-level data elements such as history of COVID-19 infection, time since vaccination, medication ordering and administration, comorbidities, other diagnosis information, and resident discharge disposition can be used in studies to assess outcomes of infections and vaccine or intervention effectiveness.^7^ Our study shows less than 10% of diagnoses among continuously present residents lacked positive SARS-CoV-2 test confirmation and supports the use of new-onset diagnosis codes to monitor COVID-19 infections in these nursing home residents. Among all residents, we identified higher rates of COVID-19 infection using the code-based definition compared with the test-based definition. This increased rate may be driven by SARS-CoV-2 testing occurring outside of the nursing home setting and clinicians diagnosing infections without laboratory confirmation. Our findings, coupled with prior work that demonstrated the nursing home EHR problem list is a tool for accurately identifying the first positive SARS-CoV-2 infection among residents from 2020 to early 2021,^8^ suggests that novel advances in nursing home data acquisition and sharing may be able to leverage diagnostic codes in the EHR to monitor nursing home COVID-19 infection rates and conduct epidemiologic studies of COVID-19.^16^

Only 24% of residents with a new-onset diagnosis code who were admitted or discharged from the facility within ±3 days of their diagnosis had a concurrent positive SARS-CoV-2 test in the nursing home, suggesting the timing of residents’ admission and discharge from the nursing home relative to COVID-19 diagnosis onset date significantly influences the PPV. This is potentially because SARS-CoV-2 testing occurs in settings outside of the nursing home. Without reliable testing information from non–nursing home sources, we cannot accurately assess the utility of diagnosis codes with an onset date near admission or discharge, which accounted for almost 40% of new-onset COVID-19 diagnoses in our time period. It is plausible that the EHR data are more complete in these time periods than laboratory test results if residents were tested elsewhere. Although sharing of information, including laboratory testing results, between nursing homes and hospitals can promote coordination of care, it is challenging to perform comprehensively.^17^ COVID-19 diagnoses included on the EHR problem list for residents who were not continuously present were more frequent among residents with a short stay, suggesting test-based surveillance might underestimate nursing home infection incidence among non–continuously present residents.

This study is subject to several limitations. Our study focused on a single corporation of nursing homes geographically clustered in the southeast that systematically documented detailed SARS-CoV-2 testing data, which may limit the generalizability of our findings. Our retrospective categorization of residents’ length of stay status may have misclassified some residents as short-stay when they had been in the nursing home more than 90 days but were discharged early in 2022. Also, because our categorization was not time-dependent, residents may have been categorized as long-stay, even if their COVID-19 event occurred while they were within the first 90 days of their stay. When comparing rates of COVID-19 infections, we used only the diagnosis codes and testing information available to the nursing home. Because we lacked additional information from registries or hospital records, the test-based rate may have undercounted infections that occurred outside of the nursing home. When assessing the concordance of positive SARS-CoV-2 tests with a corresponding diagnosis code, we lacked resident symptom data to adjudicate symptomatic and asymptomatic individuals. Therefore, we may be incorrectly assuming positive tests in asymptomatic patients should be documented as a COVID-19 infection in the nursing home EHR. Our PPV measures used a positive SARS-CoV-2 test in the nursing home for confirmation of a true COVID-19 infection. However, frequent use of antigen tests, which are less sensitive than PCR, may contribute to inaccuracies in confirmation. SARS-CoV-2 testing was conducted according to nursing home protocols similar to the CDC’s recommendations, including symptomatic and asymptomatic testing for close contacts, suggesting most cases should have been identified by tests.^18^ Some residents with unconfirmed COVID-19 infection in the EHR may have been appropriately diagnosed with COVID-19 but not tested in the nursing home, making a positive SARS-CoV-2 test an imperfect gold standard. Differences in testing and coding practices by the nursing home also may impact the variability in the PPV observed. Similar to other assessments of PPV for diagnosis codes, we did not have a gold standard to determine residents without infection, so we could not fully assess how well diagnosis codes performed at excluding COVID-19 infections.^9^

## Conclusions and Implications

Our study highlights the value of diagnosis codes for identifying COVID-19 infections in nursing homes when testing data are unavailable. Discrepancies between testing data and diagnosis codes demonstrate the value in a comprehensive approach that considers both clinical and laboratory sources of information. These findings suggest that diagnosis codes recorded on a resident’s EHR problem list are useful for ongoing COVID-19 surveillance and vaccine effectiveness studies among continuously present nursing home residents. COVID-19 diagnosis codes in EHRs can complement SARS-CoV-2 test data for accurate monitoring and evaluation of public health interventions to reduce COVID-19 and associated morbidity in the nursing home setting.

## Supplementary Material

Supp figure

Supp Table

## Figures and Tables

**Table 1:** Characteristics of Residents With Incident SARS-CoV-2–Positive Tests (n = 4876) Stratified by Presence of a Corresponding COVID-19 Diagnosis Code Within ±7 Day

Characteristic	Incident-Positive Tests*	Positive Tests With Corresponding Diagnosis Code Within ±7 Days	Positive Tests Without Corresponding Diagnosis Code Within ±7 Days	χ^2^ *P* Value	Proportion of Incident SARS-CoV-2 Tests With Corresponding Diagnosis Code
Overall	4876	4143	733		85%
Quarter of specimen collection date	n (col%)	n (col%)	n (col%)	*P*	%
Jan–Mar 2022	1870 (38)	1517 (37)	353 (48)	<.0001	81
Apr–Jun 2022	373 (8)	312 (8)	61 (8)		84
July–Sep 2022	1035 (21)	946 (23)	89 (12)		91
Oct–Dec 2022	764 (16)	676 (16)	88 (12)		88
Jan–Mar 2023	662 (14)	567 (14)	95 (13)		86
Apr–Jun 2023	172 (4)	125 (3)	47 (6)		73
Resident characteristics					
Sex					
Male	1846 (38)	1571	275	.836	85
Female	3030 (62)	2572	458		85
Age, y					
<65	679 (14)	570 (14)	109 (15)	.5726	84
65–74	1194 (24)	1021 (25)	173 (24)		86
75–84	1621 (33)	1367 (33)	254 (35)		84
>85	1382 (28)	1185 (29)	197 (27)		86
Resident length of stay in the study period					
Short stay (≤90 d)	1570 (32)	1313 (32)	257 (35)	.0719	84
Long stay (>90 d)	3306 (68)	2830 (68)	476 (65)		86
Previous COVID-19 diagnosis or positive SARS-CoV-2 test					
15–90 d prior	45 (1)	9 (0.22)	36 (5)	<.0001	20
90–180 d prior	88 (4)	64 (2)	24 (3)		73
>180 d prior	1025 (19)	810 (20)	215 (29)		79
First listed	3718 (76)	3260 (79)	458 (62)		88
Number of comorbidities^†^					
None	2407 (49)	2046 (49)	361 (49)	.4254	85
1–2	1583 (32)	1324 (32)	259 (35)		84
3–4	795 (16)	693 (17)	102 (14)		87
5–6	91 (2)	80 (2)	11 (1)		88

* Residents with incident-positive SARS-CoV-2 tests are residents who have a positive test for SARS-CoV-2 in the nursing home with no prior positive test in the nursing home in the previous 14 days. Data are shown for incident-positive tests; a resident’s characteristics may be included more than once if they had multiple incident-positive tests.

† Comorbidities were identified using ICD-10-CM diagnosis codes in the EHR and included cancer, chronic kidney disease, chronic obstructive pulmonary disease, heart conditions, immunocompromised state, chemotherapy, radiation, obesity, sickle cell disease, smoking, and diabetes.

**Table 2:** Characteristics of Residents With New-Onset ICD-10-CM Diagnoses Identified in the EHR Problem List for COVID-19 (n = 6346), Stratified by Whether the Resident Was Continuously Present in the Nursing Home for at Least 3 Days Before and After the Diagnosis Onset Date

Characteristic	All New-Onset Diagnoses*, n (col%)	Continuously Present for ± 3 Days From the Diagnosis Onset Date, n (col%)	Not Continuously Present for ± 3 Days From the Diagnosis Onset Date, n (col%)
Overall	6346 (100)	3685 (100)	2661 (100)
Quarter of diagnosis onset date			
Jan–Mar 2022	2408 (38)	1309 (36)	1099 (41)
Apr–Jun 2022	427 (7)	265 (7)	162 (6)
July–Sep 2022	1242 (20)	800 (22)	442 (17)
Oct–Dec 2022	1036 (16)	631 (17)	405 (15)
Jan–Mar 2023	996 (16)	546 (15)	450 (17)
Apr–Jun 2023	237 (4)	134 (4)	103 (4)
Resident characteristics			
Gender (missing n = 1)			
Male	2517 (40)	1344 (36)	1173 (44)
Female	3828 (60)	2340 (64)	1488 (56)
Age, y			
<65	781 (12)	528 (14)	253 (10)
65–74	1539 (24)	897 (24)	642 (24)
75–84	2118 (33)	1185 (32)	933 (35)
>85	1908 (30)	1075 (29)	833 (31)
Resident length of stay in the study period			
Short stay (≤90 d)	3100 (49)	951 (26)	2149 (81)
Long stay (>90 d)	3246 (51)	2734 (74)	512 (19)
Previous COVID-19 diagnosis or positive SARS-CoV-2 test			
15–90 d prior	23 (0.4)	20 (0.5)	3 (0.11)
90–180 d prior	71 (1)	58 (2)	13 (0.49)
>180 d prior	897 (14)	801 (22)	96 (4)
First listed	5355 (84)	2806 (76)	2549 (96)
Confirmation of infection with concordant positive test within ± 7 d	4023 (63)	3395 (92)	628 (24)

* Data are shown for new-onset diagnoses; a resident’s characteristics may be included more than once if they had multiple new-onset diagnoses in the study period.

**Table 3:** Characteristics of Residents With New-Onset ICD-10-CM Diagnoses for COVID-19 and PPV of Diagnosis Codes, Identified in the EHR Problem List (n = 3685) for Residents Continuously Present in the Nursing Home for at Least 3 Days Before and After the Diagnosis Onset Date, Stratified by the Presence of Corresponding Positive Test Within 7 Days

Characteristic	Corresponding Positive Test Within 7 Days	No Corresponding Positive Test Within 7 Days	χ^2^ *P* Value	PPV
	n (col %)	n (col %)		Estimate (95% CI)
Overall	3395	290		92% (91%–93%)
Date of onset				
Jan–Mar 2022	1210 (36)	99 (34)	<.0001	92% (91%–94%)
Apr–Jun 2022	255 (8)	10 (3)		96% (94%–99%)
July–Sep 2022	782 (23)	18 (6)		98% (97%–99%)
Oct–Dec 2022	555 (16)	76 (26)		88% (85%–91%)
Jan–Mar 2023	478 (14)	68 (23)		88% (85%–90%)
Apr–Jun 2023	115 (3)	19 (7)		86% (79%–92%)
Resident characteristics				
Sex				
Male	1245 (37)	99 (34)	.4128	93% (91%–94%)
Female	2150 (63)	190 (66)		92% (91%–93%)
Age, y				
<65	483 (14)	45 (16)	.4564	91% (89%–94%)
65–74	822 (24)	75 (26)		92% (90%–94%)
75–84	1104 (33)	81 (28)		93% (92%–95%)
>85	986 (29)	89 (31)		92% (90%–93%)
Resident length of stay in the study period				
Short stay (≤90 d)	881 (26)	70 (24)	.4985	93% (91%–94%)
Long stay (>90 d)	2514 (74)	220 (76)		92% (91%–93%)
Previous COVID-19 diagnosis or positive				
SARS-CoV-2 test				
15–90 d prior	3 (0.09)	17 (6)	<.0001	15% (0%–55%)
90–180 d prior	54 (2)	4 (1)		93% (86%–100%)
>180 d prior	737 (22)	64 (22)		92% (90%–94%)
First listed	2601 (77)	205 (71)		93% (92%–94%)
Number of comorbidities*				
None	1590 (47)	148 (51)	.0760	91% (90%–93%)
1–2	1152 (34)	94 (32)		92% (91%–94%)
3–4	587 (17)	38 (13)		94% (92%–96%)
5–6	66 (2)	10 (3)		87% (79%–95%)

Data are shown for incident-positive tests; a resident’s characteristics may be included more than once if they had multiple incident-positive tests; *P* values are for χ^2^ tests assessing differences between characteristics and presence of a corresponding positive test.

* Comorbidities assessed include cancer, chronic kidney disease, chronic obstructive pulmonary disease, heart conditions, immunocompromised state, chemotherapy, radiation, obesity, sickle cell disease, smoking, and diabetes.
