# Supplementary material for: Evaluation of COVID-19 Diagnosis Codes for Identification of SARS-CoV-2 Infections in a Nursing Home Cohort, 2022–2023
Source: J Am Med Dir Assoc. Author manuscript; Available in PMC 2025 Mar 10. (PMC11890922; doi:10.1016/j.jamda.2024.105440)
Supplement: Supp figure [file NIHMS2059008-supplement-Supp_figure.pdf]

Supplemental Figure S1. Identification of included incident-positive SARS-Cov-2 tests and new-onset COVID-19 diagnosis codes.

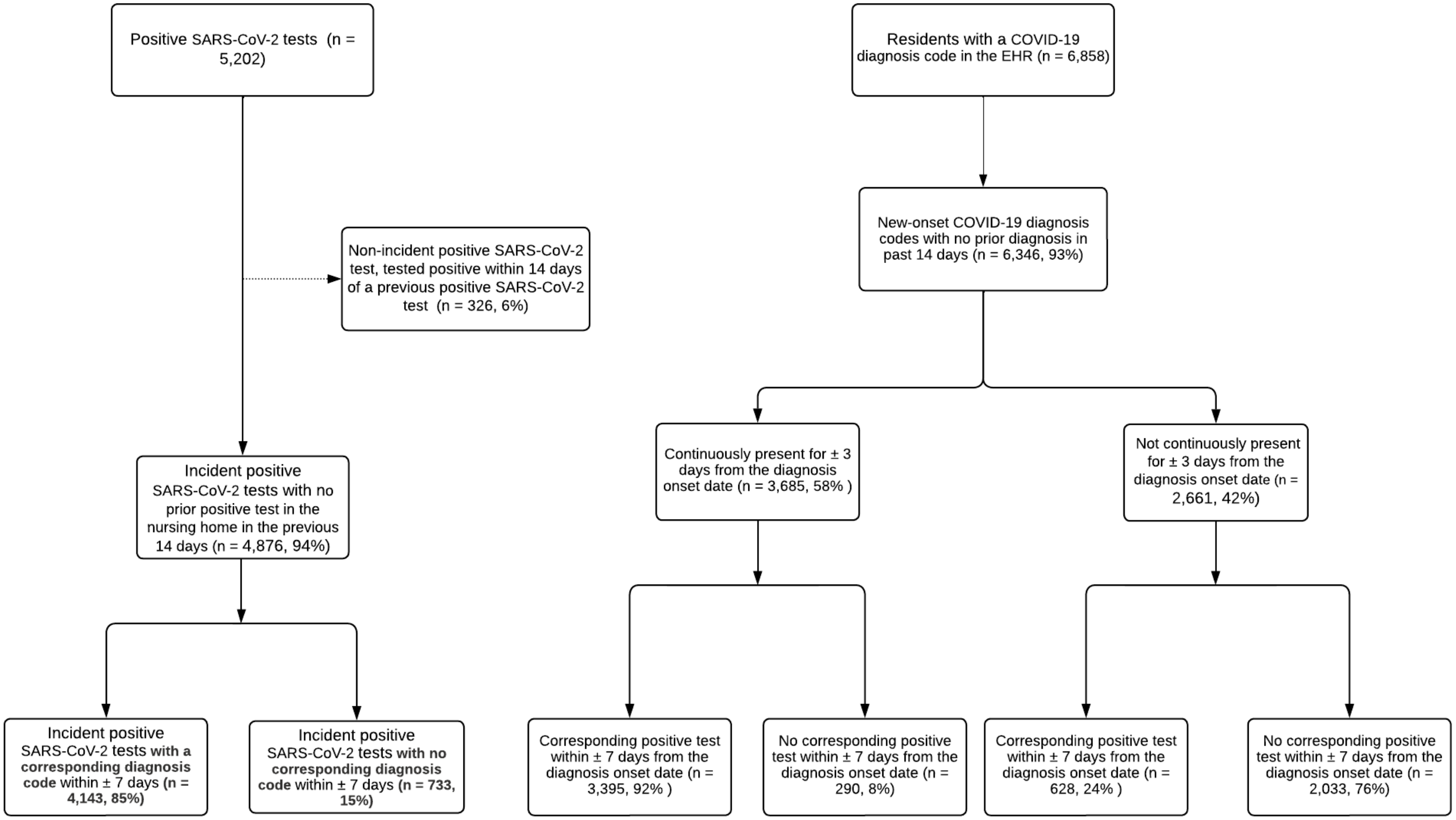

EHR: Electronic Health Record

Created in Lucidchart www.lucidchart.com
