# Supplementary material for: Evaluation of COVID-19 Diagnosis Codes for Identification of SARS-CoV-2 Infections in a Nursing Home Cohort, 2022–2023
Source: J Am Med Dir Assoc. Author manuscript; Available in PMC 2025 Mar 10. (PMC11890922; doi:10.1016/j.jamda.2024.105440)
Supplement: Supp Table [file NIHMS2059008-supplement-Supp_Table.pdf]

Supplemental Table S1. International Classification of Diseases, Tenth Revision Clinical Modification (ICD-10-CM) diagnosis codes used to identify comorbidities in the electronic health record problem list.

Comorbidities	International Classification of Diseases, Tenth Revision Clinical Modification (ICD-10-CM) diagnosis codes
Cancer	C8100, C8101, C8102, C8103, C8104, C8105, C8106, C8107, C8108, C8109, C8110, C8111, C8112, C8113, C8114, C8115, C8116, C8117, C8118, C8119, C8120, C8121, C8122, C8123, C8124, C8125, C8126, C8127, C8128, C8129, C8130, C8131, C8132, C8133, C8134, C8135, C8136, C8137, C8138, C8139, C8140, C8141, C8142, C8143, C8144, C8145, C8146, C8147, C8148, C8149, C8170, C8171, C8172, C8173, C8174, C8175, C8176, C8177, C8178, C8179, C8190, C8191, C8192, C8193, C8194, C8195, C8196, C8197, C8198, C8199, C8200, C8201, C8202, C8203, C8204, C8205, C8206, C8207, C8208, C8209, C8210, C8211, C8212, C8213, C8214, C8215, C8216, C8217, C8218, C8219, C8220, C8221, C8222, C8223, C8224, C8225, C8226, C8227, C8228, C8229, C8230, C8231, C8232, C8233, C8234, C8235, C8236, C8237, C8238, C8239, C8240, C8241, C8242, C8243, C8244, C8245, C8246, C8247, C8248, C8249, C8250, C8251, C8252, C8253, C8254, C8255, C8256, C8257, C8258, C8259, C8260, C8261, C8262, C8263, C8264, C8265, C8266, C8267, C8268, C8269, C8280, C8281, C8282, C8283, C8284, C8285, C8286, C8287, C8288, C8289, C8290, C8291, C8292, C8293, C8294, C8295, C8296, C8297, C8298, C8299, C8300, C8301, C8302, C8303, C8304, C8305, C8306, C8307, C8308, C8309, C8310, C8311, C8312, C8313, C8314, C8315, C8316, C8333, C8334, C8335, C8337, C8338, C8339, C8317, C8318, C8319, C8330, C8331, C8332, C8336, C8350, C8351, C8352, C8353, C8354, C8355, C8356, C8357, C8358, C8359, C8370, C8371, C8372, C8373, C8374, C8375, C8376, C8377, C8378, C8379, C8380, C8381, C8382, C8383, C8384, C8385, C8386, C8387, C8388, C8389, C8390, C8391, C8392, C8393, C8394, C8395, C8396, C8397, C8398, C8399, C8400, C8401, C8402, C8403, C8404, C8405, C8406, C8407, C8408, C8409, C8410, C8411, C8412, C8413, C8414, C8415, C8416, C8417, C8418, C8419, C8440, C8441, C8442, C8443, C8444, C8445, C8446, C8447, C8448, C8449, C8460, C8461, C8462, C8463, C8464, C8465, C8466, C8467, C8468, C8469, C8470, C8471, C8472, C8473, C8474, C8475, C8476, C8478, C8477, C8479, C8490, C8491, C8492, C8493, C8494, C8495, C8496, C8497, C8498, C84A0, C8499, C84A1, C84A2, 84A4, C84A3, C84A5, C84A6, C84A7, C84A8, C84A9, C84Z0, C84Z1, C84Z2, C84Z3, C84Z4, C84Z5, C84Z6, C84Z7, C84Z8, C84Z9, C8510, C8511, C8512, C8513, C8514, C8515, C8516, C8517, C8518, C8519, C8520, C8521, C8522, C8523, C8524, C8525, C8526, C8527, C8528, C8529, C8580, C8581, C8582, C8583, C8584, C8585, C8586, C8587, C8588, C8589, C8590, C8591, C8592, C8593, C8594, C8595, C8596, C8597, C8598, C8599, C860, C861, C862, C863, C864, C865, C866, C880, C884, C888, C9000, C9001, C9002, C9010 , C9012, C9020, C9022, C9030, C9032, C9100, C9102, C9110, C9112, C9130, C9132, C9140, C9142, C9150, C9152, C9160, C9162, C9190, C9192, C91A0, C91A2, C91Z0, C91Z2, C9200, C9202, C9210, C9212, C9220, C9222, C9230, C9232, C9240, C9242, C9250, C9252, C9260, C9262, C9290, C9292, C92A0, C92A2, C92Z0, C92Z2, C9300, C9302, C9310, C9312, C9330, C9332, C9390, C9392, C93Z0, C93Z2, C9400, C9402, C9420, C9422, C9430, C9432, C9440, C9442, C946, C9480, C9482, C9500, C9502, C9510, C9512,

	C9590, C9592, C9620, C9621, C9622, C9629, C964, C9660, C96A, C96Z, C969, D4622, D471, D479, D47Z1, D47Z9, D6182, D45, D474, D7581, C000, C001, C002, C003, C004, C005, C006, C008, C009, C01, C020, C021, C022, C023, C024, C028, C029, C030, C031, C039, C040, C041, C048, C049, C050, C051, C052, C058, C059, C060, C061, C062, C0680, C0689, C069, C07, C080, C081, C089, C090, C091, C098, C099, C100, C101, C102, C103, C104, C108, C109, C110, C111, C112, C113, C118, C119, C12, C130, C131, C132, C138, C139, C140, C142, C148, C153, C154, C155, C158, C159, C160, C161, C162, C163, C164, C165, C166, C168, C169, C170, C171, C172, C173, C178, C179, C180, C181, C182, C183, C184, C185, C186, C187, C188, C189, C19, C20, C210, C211, C212, C218, C220, C221, C222, C223, C224, C227, C228, C229, C23, C240, C241, C248, C249, C250, C251, C252, C253, C254, C257, C258, C259, C260, C261, C269, C300, C301, C310, C311, C312, C313, C318, C319, C320, C321, C322, C323, C328, C329, C33, C3400, C3401, C3402, C3410, C3411, C3412, C342, C3430, C3431, C3432, C3480, C3481, C3482, C3490, C3491, C3492, C37, C380, C381, C382, C383, C384, C388, C390, C399, C4000, C4001, C4002, C4010, C4011, C4012, C4020, C4021, C4022, C4030, C4031, C4032, C4080, C4081, C4082, C4090, C4091, C4092, C410, C411, C412, C413, C414, C419, C419, C430, C4310, C43111, C43112, C43121, C43122, C4320, C4321, C4322, C4330, C4331, C4339, C434, C4351, C4352, C4359, C4360, C4361, C4362, C4370, C4371, C4372, C438, C439, C4400, C4409, C44101, C441021, C441022, C441091, C441092, C44191, C441921, C441922, C441991, C441992, C44201, C44202, C44209, C44291, C44292, C44299, C44300, C44301, C44309, C44390, C44391, C44399, C4440, C4449, C44500, C44501, C44509, C44590, C44591, C44599, C44601, C44602, C44609, C44691, C44692, C44699, C44701, C44702, C44709, C44791, C44792, C44799, C4480, C4489, C4490, C4499, C450, C451, C452, C457, C459, C460, C461, C462, C463, C464, C4650, C4651, C4652, C467, C469, C469, C470, C4710, C4711, C4712, C4720, C4721, C4722, C473, C474, C475, C476, C478, C479, C480, C481, C482, C488, C490, C4910, C4911, C4912, C4920, C4921, C4922, C493, C494, C495, C496, C498, C499, C49A0, C49A1, C49A2, C49A3, C49A4, C49A5, C49A9, C4A10, C4A111, C4A112, C4A121, C4A122, C4A20, C4A21, C4A22, C4A30, C4A31, C4A39, C4A4, C4A51, C4A52, C4A59, C4A60, C4A61, C4A62, C4A70, C4A71, C4A72, C4A8, C4A9, C50011, C50012, C50019, C50021, C50022, C50029, C50111, C50112, C50119, C50121, C50122, C50129, C50211, C50212, C50219, C50221, C50222, C50229, C50311, C50312, C50319, C50321, C50322, C50329, C50411, C50412, C50419, C50421, C50422, C50429, C50511, C50512, C50519, C50521, C50522, C50529, C50611, C50612, C50619, C50621, C50622, C50629, C50811, C50812, C50819, C50821, C50822, C50829, C50911, C50912, C50919, C50921, C50922, C50929, C510, C511, C512, C518, C519, C52, C530, C531, C538, C539, C540, C541, C542, C543, C548, C549, C55, C561, C562, C569, C5700, C5701, C5702, C5710, C5711, C5712, C5720, C5721, C5722, C573, C574, C577, C578, C579, C58, C600, C601, C602, C608, C609, C61, C6200, C6201, C6202, C6210, C6211, C6212, C6290, C6291, C6292, C6300, C6301, C6302, C6310, C6311, C6312, C632, C637, C638, C639, C641, C642, C649, C651, C652, C659, C661, C662, C669, C670, C671, C672, C673, C674, C675, C676, C677, C678, C679, C680, C681, C688, C689, C6900,

	C6901, C6902, C6910, C6911, C6912, C6920, C6921, C6922, C6930, C6931, C6932, C6940, C6941, C6942, C6950, C6951, C6952, C6960, C6961, C6962, C6980, C6981, C6982, C6990, C6991, C6992, C700, C701, C709, C710, C711, C712, C713, C714, C715, C716, C717, C718, C719, C720, C721, C7220, C7221, C7222, C7230, C7231, C7232, C7240, C7241, C7242, C7250, C7259, C729, C73, C7400, C7401, C7402, C7410, C7411, C7412, C7490, C7491, C7492, C750, C751, C752, C753, C754, C755, C758, C759, C760, C761, C762, C763, C7640, C7641, C7642, C7650, C7651, C7652, C768, C770, C771, C772, C773, C774, C775, C778, C779, C7800, C7801, C7802, C781, C782, C7830, C7839, C784, C785, C786, C787, C7880, C7889, C7900, C7901, C7902, C7910, C7911, C7919, C792, C7931, C7932, C7940, C7949, C7951, C7952, C7960, C7961, C7962, C7970, C7971, C7972, C7981, C7982, C7989, C799, C7A00, C7A010, C7A011, C7A012, C7A019, C7A020, C7A021, C7A022, C7A023, C7A024, C7A025, C7A026, C7A029, C7A090, C7A091, C7A092, C7A093, C7A094, C7A095, C7A096, C7A098, C7A1, C7A012, C7A8, C7B00, C7B01, C7B02, C7B03, C7B04, C7B09, C7B1, C7B8, C800, C801, C802, C888, C889, C9620, C9629, C969, C96Z, D3701, D3702, D37030, D37031, D37032, D37039, D3704, D3705, D3709, D371, D372, D373, D374, D375, D376, D378, D379, D380, D381, D382, D383, D384, D385, D386, D390, D3910, D3911, D3912, D392, D398, D399, D400, D4010, D4011, D4012, D408, D409, D4100, D4101, D4102, D4110, D4111, D4112, D4120, D4121, D4122, D413, D414, D418, D419, D420, D421, D429, D430, D431, D432, D433, D434, D438, D439, D440, D4410, D4411, D4412, D442, D443, D444, D445, D446, D447, D449, D4709, D479, D47Z9, D480, D481, D482, D483, D484, D485, D4860, D4861, D4862, D487, D489, D408, D409, D4100, D4120, D419, D432, D433, D434, D438, D439, D4410, D443, D444, D446, D447, D4701 , D4702, D4709, D47Z9, D485, D4860, D487, D489, D490, D491, D492, D493, D494, D49511, D49512 , D49519, D4959, D496, D497, D4981, D4989, D499, Q8500, Q8501, Q8502, Q8503, Q8509,<br>D6951, D6959, D696, C9011, C9021, C9031, C9101, C9111, C9131, C9141, C9151, C9161, C9191, C91A1, C91Z1, C9201, C9211, C9221, C9231, C9241, C9251, C9291, C92A1, C92Z1, C9301, C9311, C9391, C93Z1, C9401, C9421, C9431, C9481, C9501, C9511, C9591, C9261, C9331, C9441, D472, D703, D708, D709, D72810, D72818, D72819, D729, D730, D731, D732, D733, D734, D735, D7381, D7389, D739, D7589, D759, D890, D891, D892, D893, D8940, D8941, D8942, D8943, D8949, D89810, D89811, D89812, D89813, D8982, D8989, D899, R64, R768, R769, S3600XA, S36020A, S36021A, S36029A, S36030A, S36031A, S36032A, S36039A, S3609XA.
Chemotherapy	D61810, D6481, D701, T80810, T80810A, T80810D, T80810S, Z511, Z5111, Z5112.
Radiation	Z510.
Chronic Kidney Disease	N181 , N182 , N183 , N1831, N1832, N184, N185, N186, N189, N19, N261, N269 , N29, I120, I129, I130, I1310, I1311, I132, Z9115, E0821, E0822, E0829, E0921, E0922, E0929, E1021, E1022, E1029, E1121, E1122, E1129, E1321, E1322, E1329, D631, Q618, Q619, I120, I1311, I132.

Chronic Obstructive Pulmonary Disease	J410, J411, J418, J42, J430, J431, J432, J438, J439, J440, J441, J449.
Heart Conditions	I110, I130, I132, I423, I421, I422, I428, I424, I420, I425, I429, I426, I43, I427, I509, I501, I5020, I5021, I5022, I5023, I5030, I5031, I5032, I5033, I5040, I5041, I5042, I5043, I5081, I50810, I50811, I50812, I50813, I50814, I5082, I5083, I5084, I5089, I0981, T8622, I509, I6501, I6502, I6503, I6509, I651, I6521, I6522, I6523, I6529, I658, I659, I6601, I6602, I6603, I6609, I6611, I6612, I6613, I6619, I6621, I6622, I6623, I6629, I663, I668, I669, I670, I671, I672, I673, I674, I675, I676, I677, I6781, I6782, I6783, I67841, I67848, I67850, I67858, I6789, I679, I680, I682, I688, I6900, I69010, I69011, I69012, I69013, I69014, I69015, I69018, I69019, I69020, I69021, I69022, I69023, I69028, I69031, I69032, I69033, I69034, I69039, I69041, I69042, I69043, I69044, I69049, I69051, I69052, I69053, I69054, I69059, I69061, I69062, I69063, I69064, I69065, I69069, I69090, I69091, I69092, I69093, I69098, I6910, I69110, I69111, I69112, I69113, I69114, I69115, I69118, I69119, I69120, I69121, I69122, I69123, I69128, I69131, I69132, I69133, I69134, I69139, I69141, I69142, I69143, I69144, I69149, I69151, I69152, I69153, I69154, I69159, I69161, I69162, I69163, I69164, I69165, I69169, I69190, I69191, I69192, I69193, I69198, I6920, I6921, I69210, I69211, I69212, I69213, I69214, I69215, I69218, I69219, I69220, I69221, I69222, I69223, I69228, I69231, I69232, I69233, I69234, I69239, I69241, I69242, I69243, I69244, I69249, I69251, I69252, I69253, I69254, I69259, I69261, I69262, I69263, I69264, I69265, I69269, I69290, I69291, I69292, I69293, I69298, I6930, I6931, I69310, I69311, I69312, I69313, I69314, I69315, I69318, I69319, I69320, I69321, I69322, I69323, I69328, I69331, I69332, I69333, I69334, I69339, I69341, I69342, I69343, I69344, I69349, I69351, I69352, I69353, I69354, I69359, I69361, I69362, I69363, I69364, I69365, I69369, I69390, I69391, I69392, I69393, I69398, I6980, I6981, I69810, I69811, I69812, I69813, I69814, I69815, I69818, I69819, I69820, I69821, I69822, I69823, I69828, I69831, I69832, I69833, I69834, I69839, I69841, I69842, I69843, I69844, I69849, I69851, I69852, I69853, I69854, I69859, I69861, I69862, I69863, I69864, I69865, I69869, I69890, I69891, I69892, I69893, I69898, I699, I6991, I69910, I69911, I69912, I69913, I69914, I69915, I69918, I69919, I69920, I69921, I69922, I69923, I69928, I69931, I69932, I69933, I69934, I69939, I69941, I69942, I69943, I69944, I69949, I69951, I69952, I69953, I69954, I69959, I69961, I69962, I69963, I69964, I69965, I69969, I69990, I69991, I69992, I69993, I69998, I501, I5020, I5021, I5022, I5023, I5030, I5031, I5032, I5033, I5040, I5041, I5042, I5043, I50810, I50811, I50812, I50813, I50814, I5082, I5083, I5084, I5089, I509, I402, I425, I426, I427, I428, I429, I480, I481, I4811, I4819, I482, I4820, I4821, I483, I484, I4891, I4892, I2101, I2102, I2109, I2111, I2119, I2121, I2129, I213, I214, I219, I21A1, I21A9, I220, I221, I222, I228, I229.
Immunocompromised State	Z4822, Z940, Z4821, Z941, Z953, Z954, Z945, Z4824, Z942, Z48280, Z943, Z4823, Z944, Z48290, Z9481, Z9484, Z9483, Z9482, Z48298, Z9489, Z48288, Z949, T86890, T86891, T86892, T86898, T86899, T8690, T8691, T8692, T8693, T8699, T8610, T8611, T8612, T8613, T8619, T8640, T8641, T8642, T8643, T8649, T8620, T8621, T8622, T8623, T86290, T86298, T86810, T86811, T86812, T86818, T86819, T8630, T8631, T8632, T8633, T8639,

	T8600, T8601, T8602, T8603, T8609, T86850, T86851, T86852, T86858, T86859, T865.
Obesity	E65, E6609, E661, E663, E668, E669, Z6830, Z6831, Z6832, Z6833, Z6834, Z6835, Z6836, Z6837, Z6838, Z6839, Z9884.
Sickle Cell Disease	D5740, D57411, D57412, D57419, D573, D571, D5700, D5701, D5702, D5720, D57211, D57212, D57219, D5780, D57811, D57812, D57819, D57213, D57218, D57413, D57418, D5742, D57431, D57432, D57433, D57438, D57439, D5744, D57451, D57452, D57453, D57458, D57459, D57813, D57818.
Smoking	F17200, F17201, F17203, F17208, F17209, F17210, F17211, F17213, F17218, F17219, F17220, F17221, F17223, F17228, F17229, F17290, F17291, F17293, F17298, F17299, T6511D, T6511S, T6512D, T6512S, T6513D, T6513S, T6514D, T6514S, T65211A, T65212A, T65213A, T65214A, T6521D, T6521S, T65221A, T65222A, T65223A, T65224A, T6522D, T6522S, T6523D, T6523S, T6524D, T6524S, T65291A, T65292A, T65293A, T65294A, T6591D, T6591S, T6592D, T6592S, T6593D, T6593S, T6594D, T6594S, Z87891.
Diabetes	E1011, E1021, E1022, E1029, E10311, E10319, E103211, E103212, E103213, E103219, E103291, E103292, E103293, E103299, E103311, E103312, E103313, E103319, E103391, E103392, E103393, E103399, E103411, E103412, E103413, E103419, E103491, E103492, E103493, E103499, E103511, E103512, E103513, E103519, E103521, E103522, E103523, E103529, E103531, E103532, E103533, E103539, E103541, E103542, E103543, E103549, E103551, E103552, E103553, E103559, E103591, E103592, E103593, E103599, E1036, E1037X1, E1037X2, E1037X3, E1037X9, E1039, E1040, E1041, E1042, E1043, E1044, E1049, E1051, E1052, E1059, E10610, E10618, E10620, E10621, E10622, E10628, E10630, E10638, E10641, E10649, E1065, E1069, E108, E109, E1100, E1101, E1110, E1111, E1121, E1122, E1129, E11311, E11319, E113211, E113212, E113213, E113219, E113291, E113292, E113293, E113299, E113311, E113312, E113313, E113319, E113391, E113392, E113393, E113399, E113411, E113412, E113413, E113419, E113491, E113492, E113493, E113499, E113511, E113512, E113513, E113519, E113521, E113522, E113523, E113529, E113531, E113532, E113533, E113539, E113541, E113542, E113543, E113549, E113551, E113552, E113553, E113559, E113591, E113592, E113593, E113599, E1136, E1137X1, E1137X2, E1137X3, E1137X9, E1139, E1140, E1141, E1142, E1143, E1144, E1149, E1151, E1152, E1159, E11610, E11618, E11620, E11621, E11622, E11628, E11630, E11638, E11641, E11649, E1165, E1169, E118, E119, E0800, E0801, E0810, E0811, E0821, E0822, E0829, E08311, E08319, E083211, E083212, E083213, E083219, E083291, E083292, E083293, E083299, E083311, E083312, E083313, E083319, E083391, E083392, E083393, E083399, E083411, E083412, E083413, E083419, E083491, E083492, E083493, E083499, E083511, E083512, E083513, E083519, E083521, E083522, E083523, E083529, E083531, E083532, E083533, E083539, E083541, E083542, E083543, E083549, E083551, E083552, E083553, E083559, E083591, E083592, E083593, E083599, E0836, E0837X1, E0837X2, E0837X3, E0837X9, E0839, E0840, E0841, E0842, E0843, E0844, E0849, E0851, E0852, E0859, E08610, E08618, E08620, E08621, E08622, E08628, E08630, E08638, E08641, E08649, E0865,

	E0869, E088, E089, E0900, E0901, E0910, E0911, E0921, E0922, E0929, E09311, E09319, E093211, E093212, E093213, E093219, E093291, E093292, E093293, E093299, E093311, E093312, E093313, E093319, E093391, E093392, E093393, E093399, E093411, E093412, E093413, E093419, E093491, E093492, E093493, E093499, E093511, E093512, E093513, E093519, E093521, E093522, E093523, E093529, E093531, E093532, E093533, E093539, E093541, E093542, E093543, E093549, E093551, E093552, E093553, E093559, E093591, E093592, E093593, E093599, E0936, E0937X1, E0937X2, E0937X3, E0937X9, E0939, E0940, E0941, E0942, E0943, E0944, E0949, E0951, E0952, E0959, E09610, E09618, E09620, E09621, E09622, E09628, E09630, E09638, E09641, E09649, E0965, E0969, E098, E099, E1300, E1301, E1310, E1311, E1321, E1322, E1329, E13311, E13319, E133211, E133212, E133213, E133219, E133291, E133292, E133293, E133299, E133311, E133312, E133313, E133319, E133391, E133392, E133393, E133399, E133411, E133412, E133413, E133419, E133491, E133492, E133493, E133499, E133511, E133512, E133513, E133519, E133521, E133522, E133523, E133529, E133531, E133532, E133533, E133539, E133541, E133542, E133543, E133549, E133551, E133552, E133553, E133559, E133591, E133592, E133593, E133599, E1336, E1337X1, E1337X2, E1337X3, E1337X9, E1339, E1340, E1341, E1342, E1343, E1344, E1349, E1351, E1352, E1359, E13610, E13618, E13620, E13621, E13622, E13628, E13630, E13638, E13641, E13649, E1365, E1369, E138, E139, E232.
